# Supplementary material for: Tumor reactive γδ T cells contribute to a complete response to PD-1 blockade in a Merkel cell carcinoma patient
Source: Nat Commun. 2024 Feb 6;15:1094. doi: 10.1038/s41467-024-45449-y (PMC10848161; doi:10.1038/s41467-024-45449-y)
Supplement: Supplementary file 1 — Supplementary Information [file 41467_2024_45449_MOESM1_ESM.pdf]

Supplementary Information for

Tumor reactive γδ T cells contribute to a complete response to PD-1 blockade in a Merkel cell carcinoma patient

Scott C. Lien [1,2], Dalam Ly [1], S.Y. Cindy Yang [1], Ben X. Wang [1], Derek L. Clouthier [1], Michael St. Paul [1], Ramy Gadalla [1], Babak Noamani [1], Carlos R. Garcia-Batres [1], Sarah Boross-Harmer [3], Philippe L. Bedard [3], Trevor J. Pugh [1,4,5], Anna Spreafico [3], Naoto Hirano [1,2], Albiruni R.A. Razak [3], Pamela S. Ohashi [1, 2,*]

Affiliations:

[1] Princess Margaret Cancer Centre, University Health Network, Toronto, Ontario, Canada.

[2] Department of Immunology, University of Toronto, Toronto, Ontario, Canada.

[3] Division of Medical Oncology and Haematology, Princess Margaret Cancer Centre, University Health Network, University of Toronto, Toronto, Ontario, Canada.

[4] Department of Medical Biophysics, University of Toronto, Toronto, ON, Canada.

[5] Ontario Institute for Cancer Research, Toronto, ON, Canada.

[*] Corresponding author: Pamela S. Ohashi. e-mail: pam.ohashi@uhnresearch.ca

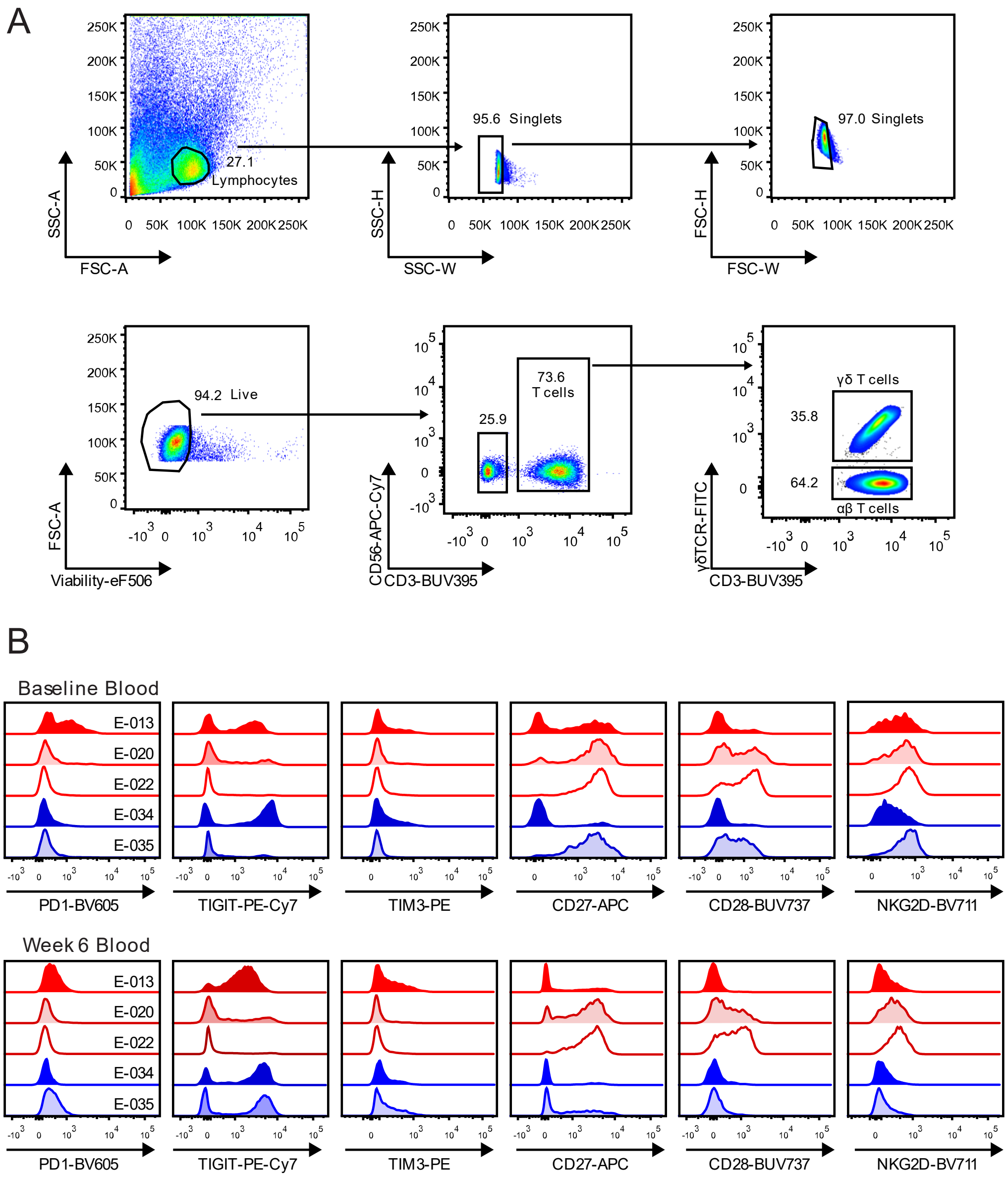

Supplementary Fig. 1. γδ T cells from patient E-013 have an activated phenotype.

Flow cytometry analysis for γδ T cells. **A**, Example of gating strategy for patient E-013 on-treatment tumor biopsy. **B**, Histogram of activation, inhibitory and co-stimulatory markers on γδ T cells from baseline and week 6 peripheral blood.

A

	TRGV	CDR3 γ	TRGJ	TRDV	CDR3 δ	TRDJ
gdTCR1-4	2	CATWDGSRSYYKKLF	1	1	CALDLGGRGPPP TDKLIF	1
gdTCR3-5	8	CATWDPYYKKLF	1	1	CALGDYVLSDKLIF	1
gdTCR3-4	2	CATWDGSRSYYKKLF	1	1	CALGDYVLSDKLIF	1

B

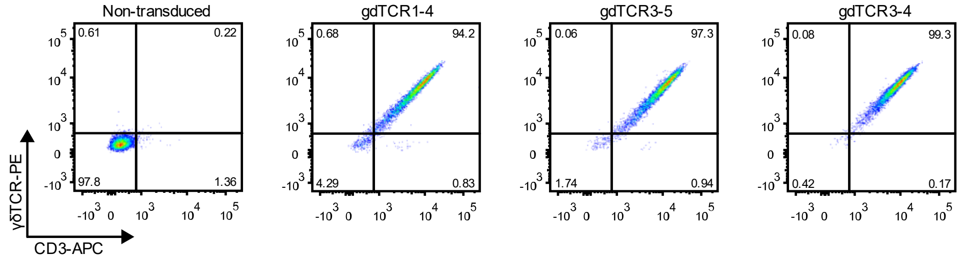

Supplementary Fig. 2. Transduced γδTCR3-4 and mis-paired control TCR are expressed on the cell surface.

A, CDR3 a.a. sequences of γδTCRs cloned into Jurkat76 cells. **B**, Cell surface expression of γδTCR and CD3 in transduced Jurkat76 cells.

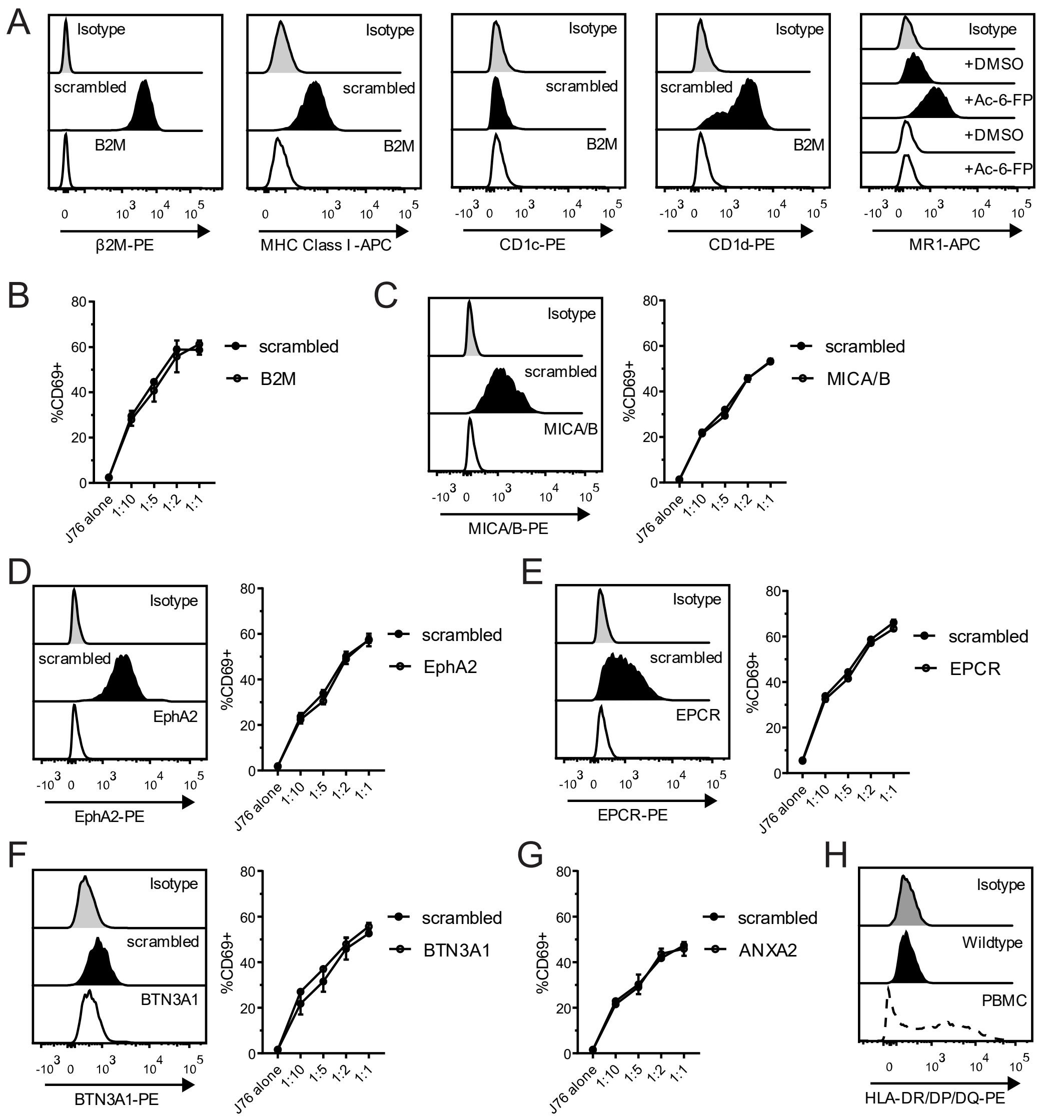

Supplementary Fig. 3. The γδTCR3-4 does not react with any known γδ T cell ligand. To examine the specificity of γδTCR3-4, various known γδ T cell ligands were knocked out of the MCC26 Merkel cell line using CRISPR technology. **A**, Cell surface expression of different antigen presentation molecules in MCC26 cells with β2M knocked out. Black histogram represents scrambled gRNA control and white histogram represents CRISPR-KO cells. **B**, γδTCR3-4 Jurkat76 cells were co-cultured with increasing number of scrambled vs. *B2M* knockout MCC26 target cells. **C**, Cell surface expression of MICA/B in MCC26 knocked out for

MICA/B (left) and co-culture of γδTCR3-4 Jurkat76 cells with *MICA/B* knockout MCC26 cells. **D**, Cell surface expression of EphA2 in MCC26 knocked out for EphA2 (left) and co-culture of γδTCR3-4 Jurkat76 cells with *EphA2* knockout MCC26 cells. **E**, Cell surface expression of EPCR in MCC26 knocked out for EPCR (left) and co-culture of γδTCR3-4 Jurkat76 cells with *EPCR* knockout MCC26 cells. **F**, Cell surface expression of BTN3A1 in MCC26 knocked out for BTN3A1 (left) and co-culture of γδTCR3-4 Jurkat76 cells with *BTN3A1* knockout MCC26 cells. **G**, Co-culture of γδTCR3-4 Jurkat76 cells with *ANXA2* knockout MCC26 cells. **H**, Cell surface expression of HLA-DR/DP/DQ in MCC26 cells (black histogram) and PBMCs (dotted line histogram). **B**-**G**, Co-culture experiments were plated in duplicate and examined in two independent experiments. Data are presented as mean values +/- SD.

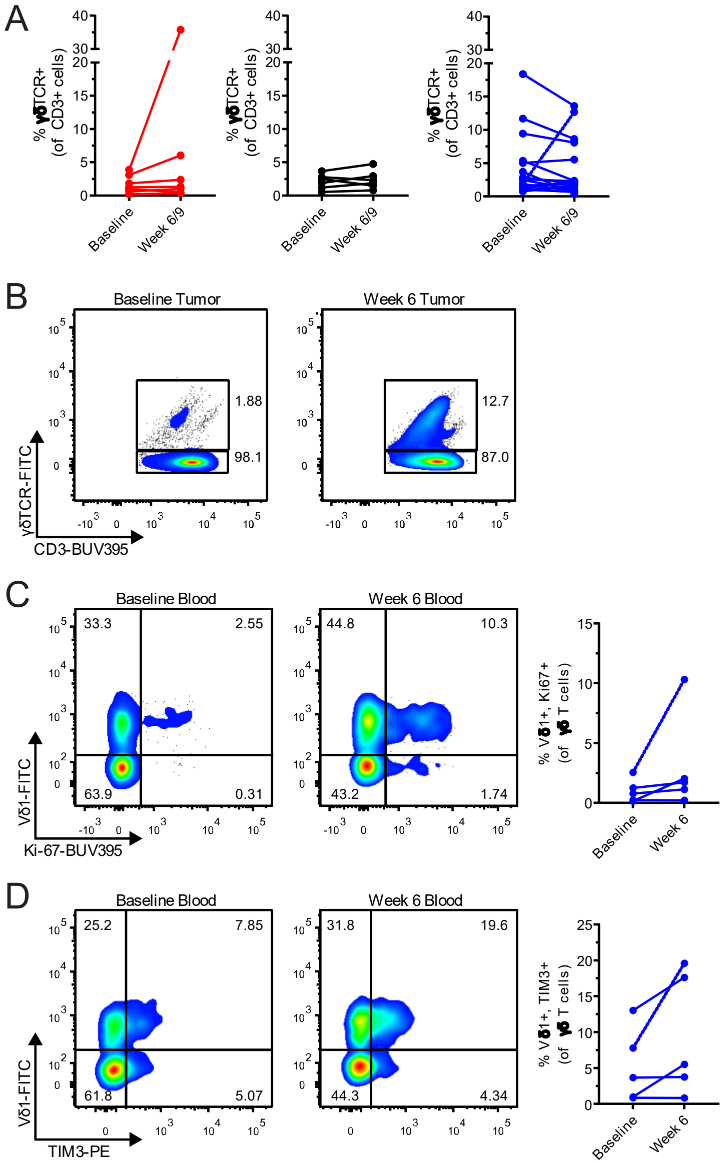

Supplementary Fig. 4. Expansion of γδ T cells in tumor of TNBC patient after pembrolizumab treatment.

A, Frequencies of γδ T cells in paired baseline and on-treatment tumor biopsies of INSPIRE patients treated with pembrolizumab. Left graph in red denotes patients with partial or complete response. The complete responder in MCC patient E-013 is also included. Middle graph in black represents patients with stable disease. Right graph in blue represents patients with progressive

disease. Dotted blue line indicates TNBC patient with six-fold expansion in the frequency of γδ T cells in the tumor. **B**, Single cell suspensions from tumor core biopsies of a TNBC patient were evaluated by flow cytometry. Samples were gated on live CD3+ cells. **C**, Flow cytometry analysis of proliferating γδ T cells from peripheral blood of TNBC patient with γδ T cell expansion in tumor (left). Summary of $Ki67^+$ Vδ1 cells in peripheral blood of five TNBC patients treated with pembrolizumab (right). **D**, Flow cytometry analysis of $V\delta1^+$ $TIM3^+$ γδ T cells from the peripheral blood of TNBC patient with γδ T cell expansion in tumor (left). Summary of $TIM3^+$ Vδ1 cells in peripheral blood of five TNBC patients treated with pembrolizumab (right).

Patient ID	Sex	Age Range	Stage at diagnosis	MCPyV Status	RECIST 1.1 Best overall response	Overall Survival (months)	Reason off trial	Biopsy Site	Prior therapies
E-013	Male	80-84	3a	Positive	CR	61	Intercurrent illness	Chest lesion with skin	Radiation
E-020	Male	70-74	2a	Positive	PR	25.3	Clinical Progression	Right supraclav mass	Surgical excision, radiation
E-022	Male	70-74	3	Positive	PR	19	Toxicity (Pneumonitis)	Segment 5 of liver	Radiation, surgical excision
E-033	Male	65-69	2a	Positive	PD	2.4	Clinical Progression	Left oxillary LN	Surgical excision
E-034	Female	60-64	2a	Positive	PD	5.8	Clinical Progression	R neck LN	Surgical excision, radiation, carboplatin/etoposide
E-035	Male	50-54	3b	Positive	PD	19.3	Clinical Progression	R leg medial (below knee) cutaneous	None

Supplementary Table 1. Clinical characteristics of Merkel cell carcinoma patients

Fluorochrome	Marker	Vendor	Cat #	Clone	Dilution
FITC	gdTCR	eBioscience	11-9959-42	B1.1	1:50
PerCP	CD8a	Biolegend	301030	RPA-T8	1:50
PE	4-1BB	eBioscience	12-1379-42	4B4-1	1:100
PE-Cy7	TIGIT	eBioscience	25-9500-42	MBSA43	1:50
eFluor660	CTLA4 (surface)	eBioscience	50-1529-42	14D3	1:100
Alexa700	CD4	eBioscience	56-0048-42	OKT4	1:100
Alexa700	CD19	eBioscience	56-0199-42	HIB19	1:100
APC-Cy7	CD56	Biolegend	318332	HCD56	1:100
eFluor460	PD-L1	eBioscience	48-5983-42	MIH1	1:50
eFluor506	Viability	eBioscience	65-0866-18		1:800
BV605	PD-1	Biolegend	329924	EH12.2H7	1:100
BUV395	CD3	BD	563546	UCHT1	1:25

Supplementary Table 2. Antibody clone and dilutions used for T cell activation/exhaustion flow cytometry panel

**Fluorochrome**	**Marker**	**Vendor**	**Cat #**	**Clone**	**Dilution**
FITC	Vδ1	Thermo	TCR2730	TS8.2	8:100
PerCP	Vδ2	Biolegend	331410	B6	2:100
PE	TIM3	R&D	FAB2365P	344823	1:100
PE-Dazzle594	DNAM1	Biolegend	338317	11A8	1:100
PE-Cy7	LAG3	eBio	25-2239-42	3DS223H	1:100
APC	CD27	Biolegend	302810	O323	1:100
Alexa700	CD3	BD	561027	UCHT-1	1:100
APC-H7	CD8	BD	560179	SK1	1:100
BV421	CD30	BD	566253	BerH8	1:100
eF506	Viability	eBio	65-0866-18	NA	1:800
BV605	PD-1	Biolegend	329924	EH12.2H7	1:100
BV711	NKG2D	BD	563688	1D11	1:100
BV785	Streptavidin	Biolegend	405249	NA	0.5:100
BUV395	Ki67	BD	564071	B56	2:100
BUV486	CD4	BD	564651	SK3	1:100
BUV737	CD28	BD	564438	28.2	2:100
Biotinylated	Pan-γδ TCR	Miltenyi	130-096-862	11F2	16:100

Supplementary Table 3. Antibody clone and dilutions used for γδ T cell flow cytometry panel

Fluorochrome	Marker	Vendor	Cat #	Clone	Dilution
FITC	CD8	eBioscience	11-0088-42	RPA-T8	1:100
PE	γδ TCR	eBioscience	12-9959-42	B1.1	2:100
PE-Cy7	CD3	eBioscience	25-0038-42	UCHT1	1:100
APC	CD4	eBioscience	17-0049-42	RPA-T4	1:100
eF506	Viability	eBioscience	65-0866-18	NA	1:800
BV711	CD56	BD	563169	NCAM16	1:100

Supplementary Table 4. Antibody clone and dilutions used for γδ T cell FACS sorting panel

Fluorochrome	Marker	Vendor	Cat #	Clone	Dilution
PE	CD3	BD	555333	UCHT1	5:100
APC	CD69	Biolegend	310910	FN50	2:100
eF506	Viability	eBioscience	65-0866-18	NA	1:800

Supplementary Table 5. Antibody clone and dilutions used for transduced Jurkat γδTCR and cancer cell line co-culture assay

Target	#	Sequence
scrambled	1	GCACUACCAGAGCUAACUCA
B2M	1	GAGUAGCGCGAGCACAGCUA
	2	ACUCACGCUGGAUAGCCUCC
MICA/B	1	GGCAAAGCCCCAGGGACAGU
	2	GCUAUGACAGGCAGAAACGC
	3	GUCCUCCAGAGCUCAGACCU
EphA2	1	CAUGAACUACACCUUCACCG
	2	UCACGGAGAAACCCUCGGUG
	3	UCACGGAGAAACCCUCGGUG
EPCR	1	UCCGCGACCCCUAUCACGUG
	2	GGGACACCUAACGCACGUGC
	3	AAGCCGCUCCUACACUUCGC
BTN3A1	1	ACCAUCAGAAGUUCCCUCCU
	2	GGCACUUACGAGAUGCAUAC
ANXA2	1	CAGCCAUCAAGACCAAAGGU
	2	ACAGGGGCUGGGAACCGACG

Supplementary Table 6. CRISPR guides used for knocking out γδ T cell ligands in MCC26 Merkel cancer cell line.
