# Supplementary material for: Tumor reactive γδ T cells contribute to a complete response to PD-1 blockade in a Merkel cell carcinoma patient
Source: Nat Commun. 2024 Feb 6;15:1094. doi: 10.1038/s41467-024-45449-y (PMC10848161; doi:10.1038/s41467-024-45449-y)
Supplement: Supplementary file 3 — Description of Additional Supplementary Files [file 41467_2024_45449_MOESM3_ESM.pdf]

Description of Additional Supplementary Files

File Name: Supplementary Data 1

Description: scRNAseq count matrix and γδ TCR contigs
