# Supplementary material for: Tumor reactive γδ T cells contribute to a complete response to PD-1 blockade in a Merkel cell carcinoma patient
Source: Nat Commun. 2024 Feb 6;15:1094. doi: 10.1038/s41467-024-45449-y (PMC10848161; doi:10.1038/s41467-024-45449-y)
Supplement: Supplementary file 5 — Reporting Summary [file 41467_2024_45449_MOESM5_ESM.pdf]

nature portfolio

Corresponding author(s): Pamela Ohashi

Last updated by author(s): Nov 13, 2023

Reporting Summary

Nature Portfolio wishes to improve the reproducibility of the work that we publish. This form provides structure for consistency and transparency in reporting. For further information on Nature Portfolio policies, see our Editorial Policies and the Editorial Policy Checklist.

Statistics

For all statistical analyses, confirm that the following items are present in the figure legend, table legend, main text, or Methods section.

n/a	Confirmed	
☐	☒	The exact sample size (*n*) for each experimental group/condition, given as a discrete number and unit of measurement
☐	☒	A statement on whether measurements were taken from distinct samples or whether the same sample was measured repeatedly
☐	☒	The statistical test(s) used AND whether they are one- or two-sided<br>*Only common tests should be described solely by name; describe more complex techniques in the Methods section.*
☒	☐	A description of all covariates tested
☐	☒	A description of any assumptions or corrections, such as tests of normality and adjustment for multiple comparisons
☐	☒	A full description of the statistical parameters including central tendency (e.g. means) or other basic estimates (e.g. regression coefficient) AND variation (e.g. standard deviation) or associated estimates of uncertainty (e.g. confidence intervals)
☐	☒	For null hypothesis testing, the test statistic (e.g. *F*, *t*, *r*) with confidence intervals, effect sizes, degrees of freedom and *P* value noted<br>*Give P values as exact values whenever suitable.*
☒	☐	For Bayesian analysis, information on the choice of priors and Markov chain Monte Carlo settings
☒	☐	For hierarchical and complex designs, identification of the appropriate level for tests and full reporting of outcomes
☒	☐	Estimates of effect sizes (e.g. Cohen's *d*, Pearson's *r*), indicating how they were calculated

Our web collection on statistics for biologists contains articles on many of the points above.

Software and code

Policy information about availability of computer code

Data collection	Flow cytometry data was collected on a BD LSR Fortessa with BD FACSDiva. TCR sequencing data was collected by Adaptive Biotechnologies immunoSEQ service and data was exported for analysis. scRNA sequencing data was collected on an Illumina NovaSeq600 by the Princess Margaret Genomic Centre in Toronto, Canada.
Data analysis	Flow Cytometry: FlowJo (v10)<br>TCR Analysis: immunarch v(0.6.9)<br>scRNAseq: Seurat (v4.1.1), scRepertoire (v1.6.0), SingleCellExperiment (v 1.18.0), dittoSeq (v1.9.1), slingshot (v2.4.0), EnhancedVolcano (v1.14.0)<br>General: R (v4.2.0), GraphPad Prism (v7)

For manuscripts utilizing custom algorithms or software that are central to the research but not yet described in published literature, software must be made available to editors and reviewers. We strongly encourage code deposition in a community repository (e.g. GitHub). See the Nature Portfolio guidelines for submitting code & software for further information.

Data

Policy information about availability of data

All manuscripts must include a data availability statement. This statement should provide the following information, where applicable:

- Accession codes, unique identifiers, or web links for publicly available datasets
- A description of any restrictions on data availability
- For clinical datasets or third party data, please ensure that the statement adheres to our policy

The single cell gene expression count matrix and V(D)J calls generated in this study are available in the Supplementary Data file. The raw scRNAseq data are protected and are not available due to data privacy laws. The bulk TCR γ and δ sequencing data generated in this study are available in the immuneACCESS database (https://doi.org/10.21417/SCL2023NC). Source data are provided as a Source Data file in this paper.

Research involving human participants, their data, or biological material

Policy information about studies with human participants or human data. See also policy information about sex, gender (identity/presentation), and sexual orientation and race, ethnicity and racism.

Reporting on sex and gender	Sex-based analysis was not performed in our study because there were five male patients and one female patient. This skew reflects the higher incidence of Merkel cell carcinoma in males. Information of patient gender was not collected.
Reporting on race, ethnicity, or other socially relevant groupings	Data on race, ethnicity and other socially relevant groupings were not collected in this study.
Population characteristics	Patients greater than 18 years old with advanced solid tumors, and available archived or baseline tumor tissue, provided informed consent were enrolled in INSPIRE. Six Merkel cell carcinoma patients were enrolled into a sub-cohort of Mixed Advanced Solid Tumors. Participants were required to have measurable disease, ECOG status 0-1, and adequate organ function. Participants could not have had prior anti-PD1/PDL1/PDL2 therapy or active immunodeficiency, auto-immune disease, pneumonitis, active tuberculosis, or active central nervous system metastases. Patient age ranged from 52 to 81 years old (median 68 years).
Recruitment	Patients were recruited at clinics in Princess Margaret Cancer Centre (University Health Network), a research hospital in Toronto, Ontario. As patients were expected to allow on-treatment biopsies and provide research blood samples, the recruited population likely represented on average a healthier and better prognosis group than an average clinical presentation off study. This self-selection bias could result in altered effect sizes of prognostic biomarkers such as changes in immune infiltration and TCR repertoire.
Ethics oversight	The clinical trial was approved by the Research Ethics Board at University Health Network in Toronto, Canada. Written informed consent was given by all patients.

Note that full information on the approval of the study protocol must also be provided in the manuscript.

Field-specific reporting

Please select the one below that is the best fit for your research. If you are not sure, read the appropriate sections before making your selection.

☒ Life sciences ☐ Behavioural & social sciences ☐ Ecological, evolutionary & environmental sciences

For a reference copy of the document with all sections, see nature.com/documents/nr-reporting-summary-flat.pdf

Life sciences study design

All studies must disclose on these points even when the disclosure is negative.

Sample size	No sample size calculation was performed. Sample size was chosen based on number of Merkel cell carcinoma patients that were enrolled in trial.
Data exclusions	For flow cytometry of on-treatment core biopsies, in one patient, there was not enough material and in another patient, they progressed prior to the on-treatment biopsy. For flow cytometry of blood and TCR sequencing analysis, one patient progressed and was taken off trial prior to week 6.
Replication	No replication was performed on samples with patient blood or tumor biopsies due to limited biospecimen availability. In vitro assays were replicated in two independent experiments.
Randomization	There was no randomization in this study.
Blinding	Initial immunophenotyping flow cytometry panels were performed on patient blood and tumor biopsies without knowledge of patient outcomes. Later on, γδ T cell flow cytometry, TCR sequencing and scRNA sequencing were performed with knowledge of patient outcome.

Blinding was not applicable for these subsequent experiments, as the focus shifted towards targeted investigations where knowledge of patient outcomes was important for detailed understanding of the phenotypic and functional attributes of γδ T cells.

Reporting for specific materials, systems and methods

We require information from authors about some types of materials, experimental systems and methods used in many studies. Here, indicate whether each material, system or method listed is relevant to your study. If you are not sure if a list item applies to your research, read the appropriate section before selecting a response.

Materials & experimental systems

n/a	Involved in the study	
☐	☒	Antibodies
☐	☒	Eukaryotic cell lines
☒	☐	Palaeontology and archaeology
☒	☐	Animals and other organisms
☐	☒	Clinical data
☒	☐	Dual use research of concern
☒	☐	Plants

Methods

n/a	Involved in the study	
☒	☐	ChIP-seq
☐	☒	Flow cytometry
☒	☐	MRI-based neuroimaging

Antibodies

Antibodies used

The following antibodies were used for flow cytometry analysis or FACS sorting:
gdTCR-FITC (1:50, clone B1.1, eBioscience catalogue # 11-9959-42)
CD8a-PerCP (1:50, clone RPA-T8, Biolegend catalogue # 301030)
4-1BB-PE (1:100, clone 4B4-1, eBioscience catalogue # 12-1379-42)
TIGIT-PE-Cy7 (1:50, clone MBSA43, eBioscience catalogue # 25-9500-42)
CTLA4 (surface)-eFluor660 (1:100, clone 14D3, eBioscience catalogue # 50-1529-42)
CD4-Alexa700 (1:100, clone OKT4, eBioscience catalogue # 56-0048-42)
CD19-Alexa700 (1:100, clone HIB19, eBioscience catalogue # 56-0199-42)
CD56-APC-Cy7 (1:100, clone HCD56, Biolegend catalogue # 318332)
PD-L1-eFluor460 (1:50, clone MIH1, eBioscience catalogue # 48-5983-42)
PD-1-BV605 (1:100, clone EH12.2H7, Biolegend catalogue # 329924)
CD3-BUV395 (1:25, clone UCHT1, BD catalogue # 563546)
Vδ1-FITC (8:100, clone TS8.2, Thermo catalogue # TCR2730)
Vδ2-PerCP (2:100, clone B6, Biolegend catalogue # 331410)
TIM3-PE (1:100, clone 344823, R&D catalogue # FAB2365P)
DNAM1-PE-Dazzle594 (1:100, clone 11A8, Biolegend catalogue # 338317)
LAG3-PE-Cy7 (1:100, clone 3DS223H, eBio catalogue # 25-2239-42)
CD27-APC (1:100, clone O323, Biolegend catalogue # 302810)
CD3-Alexa700 (1:100, clone UCHT-1, BD catalogue # 561027)
CD8-APC-H7 (1:100, clone SK1, BD catalogue # 560179)
CD30-BV421 (1:100, clone BerH8, BD catalogue # 566253)
PD-1-BV605 (1:100, clone EH12.2H7, Biolegend catalogue # 329924)
NKG2D-BV711 (1:100, clone 1D11, BD catalogue # 563688)
Streptavidin-BV785 (0.5:100, Biolegend catalogue # 405249)
Ki67-BUV395 (2:100, clone B56, BD catalogue # 564071)
CD4-BUV486 (1:100, clone SK3, BD catalogue # 564651)
CD28-BUV737 (2:100, clone 28.2, BD catalogue # 564438)
CD8-FITC (1:100, clone RPA-T8, eBioscience catalogue # 11-0088-42)
γδ TCR-PE (2:100, clone B1.1, eBioscience catalogue # 12-9959-42)
CD3-PE-Cy7 (1:100, clone UCHT1, eBioscience catalogue # 25-0038-42)
CD4-APC (1:100, clone RPA-T4, eBioscience catalogue # 17-0049-42)
CD56-BV711 (1:100, clone NCAM16, BD catalogue # 563169)
CD3-PE (5:100, clone UCHT1, BD catalogue # 555333)
CD69-APC (2:100, clone FN50, Biolegend catalogue # 310910)
Pan-γδ TCR-Biotinylated (16:100, clone 11F2, Miltenyi catalogue # 130-096-862)

Validation

Antibodies used for flow cytometry were titrated for optimal staining intensity using healthy donor PBMCs. Validation against IgG isotype control staining human cells for flow cytometry can be found on manufacturer's website.

Eukaryotic cell lines

Policy information about cell lines and Sex and Gender in Research

Cell line source(s)

The following Merkel cell carcinoma lines were purchased from Sigma-Aldrich as part of the European Collection of Authenticated Cell Cultures: MCC14/2 (Catalog #10092303), MCC26 (Catalog #10092304), MS-1 (Catalog #09111802). The cell line CaOV3 (Catalog #HTB-75) and Human Umbilical Vein Endothelial Cells (Catalog #CRL1730) were purchased from the American Type Culture Collection. The cell lines OVCAR3, H929 and A375 were gifts from the Mak lab (Princess Margaet

	Cancer Centre, Toronto, Canada). The Jurkat 76 cells were a gift from Dr. M. Heemskerk, (Leiden University Medical Center, Leiden, the Netherlands) The melanoma cell lines 624mel and 888mel were gifts from Dr. S. Rosenberg (National Cancer Institute).
Authentication	None of the cell lines used were authenticated.
Mycoplasma contamination	Cell lines were not tested for mycoplasma contamination.
Commonly misidentified lines (See ICLAC register)	No commonly misidentified cell lines were used.

Clinical data

Policy information about clinical studies

All manuscripts should comply with the ICMJE guidelines for publication of clinical research and a completed CONSORT checklist must be included with all submissions.

Clinical trial registration	NCT02644369
Study protocol	Trial protocol can be found in our previously published report: Yang, S. Y. et al. Pan-cancer analysis of longitudinal metastatic tumors reveals genomic alterations and immune landscape dynamics associated with pembrolizumab sensitivity. Nat Commun 12, (2021).
Data collection	Patient recruitment and data collection was conducted at the Princess Margaret Cancer Centre from March 21, 2016 to May 9, 2018. Data collection cutoff was December 6, 2021.
Outcomes	Primary outcomes were changes in genomic and immune biomarkers measured in the blood and tumor pre-treatment, on-treatment and at progression. Genomic and immune biomarkers were measured by TCR sequencing and flow cytometry. Survival outcomes of the study was defined by progression free survival and overall survival. Clinical outcomes was determined by RECIST 1.1 criteria.

Plants

Seed stocks	*Report on the source of all seed stocks or other plant material used. If applicable, state the seed stock centre and catalogue number. If plant specimens were collected from the field, describe the collection location, date and sampling procedures.*
Novel plant genotypes	*Describe the methods by which all novel plant genotypes were produced. This includes those generated by transgenic approaches, gene editing, chemical/radiation-based mutagenesis and hybridization. For transgenic lines, describe the transformation method, the number of independent lines analyzed and the generation upon which experiments were performed. For gene-edited lines, describe the editor used, the endogenous sequence targeted for editing, the targeting guide RNA sequence (if applicable) and how the editor was applied.*
Authentication	*Describe any authentication procedures for each seed stock used or novel genotype generated. Describe any experiments used to assess the effect of a mutation and, where applicable, how potential secondary effects (e.g. second site T-DNA insertions, mosiacism, off-target gene editing) were examined.*

Flow Cytometry

Plots

Confirm that:

☒ The axis labels state the marker and fluorochrome used (e.g. CD4-FITC).

☒ The axis scales are clearly visible. Include numbers along axes only for bottom left plot of group (a 'group' is an analysis of identical markers).

☒ All plots are contour plots with outliers or pseudocolor plots.

☒ A numerical value for number of cells or percentage (with statistics) is provided.

Methodology

Sample preparation	Pooled tumor core biopsies or tissue samples were minced into 2-4mm3 fragments and digested with the gentle MACS dissociator (Miltenyi, Catalog #130–093-235) and the human tumor dissociation kit (Miltenyi, Catalog #130–095-929).
Instrument	Flow cytometry data was collected with a five laser BD LSR Fortessa.
Software	BD FACSDiva was used to acquire data and TreeStar FlowJo (v10) was used for analysis.
Cell population abundance	Prior to cell sorting, γδ T cells accounted for 1.12-11.3% of the live CD3+ lymphocyte gate. After FACS sorting, γδ T cells accounted for 78.1-99.3% of live CD3+ lymphocytes. Purity was determined by performing flow cytometry on the post-sort fraction.

Gating strategy

In Figure 1A:
1) FSC-A vs SSC-A gated on lymphocytes
2) SSC-H vs SSC-W gated on singlets
3) FSC-H vs FSC-W gated on singlets
4) FSC-A vs Viability-eF506 gated on live cells
5) CD56-APC-Cy7 vs CD3-BUV395 gated on CD3+ T cells
6) γδ TCR-FITC vs CD3-BUV395 gated on γδ TCR+ and CD3+ cells

In Figure 1C:
1) FSC-A vs SSC-A gated on lymphocytes
2) SSC-H vs SSC-W gated on singlets
3) FSC-H vs FSC-W gated on singlets
4) FSC-A vs Viability-eF506 gated on live cells
5) CD56-APC-Cy7 vs CD3-BUV395 gated on CD3+ T cells
6a) γδ TCR-FITC vs CD3-BUV395 gated on γδ TCR+ and CD3+ cells
7a) PD1-BV605 vs TIGIT-PECy7 gated on γδ TCR+
7b) CD8-PerCP vs CD4-AF700 gated on CD3+
8b) PD1-BV605 vs TIGIT-PECy7 gated on CD8+ and CD4+

In Figure 2B and D:
1) FSC-A vs SSC-A gated on lymphocytes
2) SSC-H vs SSC-W gated on singlets
3) FSC-H vs FSC-W gated on singlets
4) FSC-A vs Viability-eF506 gated on live cells
5) γδ TCR-BV786 vs CD3-AF700 gated on γδ TCR+ and CD3+ cells
6a) Vd1-FITC vs Vd2-PerCP gated on γδ TCR+ cells
6b) Vd1-FITC vs Ki67-BUV395 gated on γδ TCR+ cells

In Figure 2F:
1) FSC-A vs SSC-A gated on lymphocytes
2) SSC-H vs SSC-W gated on singlets
3) FSC-H vs FSC-W gated on singlets
4) FSC-A vs Viability-eF506 gated on live cells
5) γδ TCR-BV786 vs CD3-AF700 gated on γδ TCR+ and CD3+ cells
6a) PD1-BV605 vs TIM3-PE gated on γδ TCR+
6b) CD8-APC-H7 vs CD4-BUV486
7b)PD1-BV605 vs TIM3-PE gated on CD8+ and CD4+

☒ Tick this box to confirm that a figure exemplifying the gating strategy is provided in the Supplementary Information.
